# Circulating mircoRNA-21 as a predictor for vascular restenosis after interventional therapy in patients with lower extremity arterial occlusive disease

**DOI:** 10.1042/BSR20160502

**Published:** 2017-04-20

**Authors:** Bo Zhang, Ye Yao, Qing-Feng Sun, Si-qi Liu, Bao Jing, Chao Yuan, Xin-Yu Liu, Tong Jiao, Hao-cheng Li, Hai-Yang Wang

**Affiliations:** Department of Vascular Surgery, The First Affiliated Hospital of Harbin Medical University, Harbin 150001, P.R. China

**Keywords:** Interventional therapy, Lower extremity arterial occlusive disease, Sensitivity, Specificity, the First Affiliated Hospital of Harbin Medical University, Vascular restenosis

## Abstract

The present study was designed to investigate the role of circulating miRNA-21 (*miR-21*) in vascular restenosis of lower extremity arterial occlusive disease (LEAOD) patients after interventional therapy. A total of 412 LEAOD patients were enrolled randomly in the present study. According to computed tomography angiography (CTA) and ankle-brachial index (ABI), patients were assigned into the restenosis group and the non-restenosis group. *miR-21* expression was detected with quantitative real-time PCR (qRT-PCR) before and after patients underwent interventional therapy. A follow-up period of 6 months was achieved. A receiver operating characteristic (ROC) curve was drawn and the area under the curve (AUC) was calculated to assess the predictive value of *miR-21* in vascular restenosis. Patients were older in the restenosis group than in the non-restenosis group. The percentages of patients with diabetes and hypertension were higher in the restenosis group than in the non-restenosis group, and the Fontaine stage exhibited a significant difference between the two groups. *miR-21* expression was higher in the restenosis group than in the non-restenosis group. *miR-21* expression level was related to age, diabetes and hypertension in the restenosis group. Using *miR-21* to predict vascular restenosis yielded an AUC of 0.938 (95% confidence interval (CI): 0.898–0.977), with Youden index of 0.817, sensitivity of 83.5% and specificity of 98.2%. Logistic regression analysis revealed that diabetes and *miR-21* expression were the major risk factors for vascular restenosis of LEAOD. *miR-21* can be used as a predictive indicator for vascular restenosis of LEAOD after interventional therapy.

## Introduction

Lower extremity arterial occlusive disease (LEAOD) is the most common cause of systemic atherosclerosis in the limbs [1]. The growth of secondary thrombosis or atherosclerotic material can result in stenosis or occlusion of the arterial lumen, clinically presenting as a limb blood circulation disorder with which patients can easily suffer gangrene or ulcers [2]. LEAOD mainly occurs in patients over the age of 40, and the incidence was higher in men than in women, ascending with increased age while the incidence of claudication in women aged over 50 years approaches that of men [3]. LEAOD usually causes embolism, thrombus formation or stenosis, leading to acute or chronic ischaemia of the lower limbs. Percutaneous transluminal angioplasty (PTA) serves as the major interventional treatment for LEAOD, which gradually dilates the location of the stenosis or occlusion for recanalization using an expanding balloon catheter [4]. Although promising therapies have been suggested, there has emerged a high incidence of vascular restenosis that will limit the effectiveness of these therapeutic procedures [5].

miRNAs are endogeneous small non-coding RNAs of ~23 nts in length [6]. Patients with vascular diseases always have certain patterns of circulating miRNA levels in the early disease stages [7], and a recent study showed that different miRNAs in vascular biology can directly or indirectly function in post-transcriptional regulation of fundamental genes involved in vascular remodelling, thus, designated miRNAs can serve as potential biomarkers or promising drug targets [8]. Santulli et al. have demonstrated that a miRNA-based approach developed to inhibit proliferative vascular smooth muscle cells can prevent restenosis, while selectively promoting re-endothelialization and preserving endothelial cell function [9]. Specifically, controlling *miR-126* expression contributes to the inhibition of restenosis and thrombosis, thus preserving endothelial function [10]. A report has shown the fundamental role of multiple miRNAs including *miR-21*, *miR-146* and *miR142-3p* in restenosis due to their aberrant expression in stented pig arteries [11]. Located on chromosome 17, *miR-21* is a subtype of the miRNA family and has its own promoter region [12]. Overexpression of *miR-21* is shown to be involved in a variety of pathological conditions including cancer and cardiovascular disease, and it can affect the development of atherosclerosis through regulating specific cells [13–15]. Importantly, antisense knockdown of *miR-21*, which is increased after vascular injury, can prevent the neointimal lesion formation in response to balloon injury of carotid arteries [16].

Therefore, the present study aims to investigate the role of circulating *miR-21* in vascular restenosis of LEAOD after interventional therapy.

## Materials and methods

### Ethics statement

All experimental procedures were approved by the Ethics Committee of the First Affiliated Hospital of Harbin Medical University. Written informed consent was obtained from all the patients.

### Study participants

Data from a total of 412 patients diagnosed with LEAOD at the First Affiliated Hospital of Harbin Medical University between January 2010 and March 2014 were collected, including 307 males and 105 females, between 33–89 years old, with a mean age of 58.49 ± 11.61 years, average body mass index (BMI) of 22.83 ± 2.50 kg/m^2^. And the duration of the disease was between 0.8–7.3 years and 3.07 ± 0.48 on average. The clinical features of LEAOD patients staging by Fontaine were as follows: 0 case of stage I (asymptomatic); 174 cases of stage II (intermittent claudication); 207 cases of stage III (rest pain) and 25 cases of stage IV (severe ischaemia, anabrosis and necrosis). Six cases of ineffective patients (one case in stage III and five cases in stage IV) were excluded in the study, and the remaining 406 cases were assigned into restenosis group and non-restenosis group after six months of interventional therapy according to computed tomography angiography (CTA) and ankle-brachial index (ABI). Inclusion criteria: (i) pre-operative diagnosis was in accordance with the American College of Cardiology (ACC/AHA) diagnostic criteria in 2005; (ii) ABI <0.9, lower extremity arterial stenosis as tested by CTA ≥50%; (iii) evident intermittent claudication of lower limbs, rest pain, anabrosis or gangrene; (iv) patients were over 18 years old and were co-operative in therapeutic observation. Exclusion criteria: (i) patients with congenital diseases, hereditary disease, autoimmune disease or psychiatric disease; (ii) patients diagnosed with malignant tumour diseases; (iii) pregnancy or women in lactation; (iv) patients with no ability to receive antiplatelet drugs continuously after surgery.

### Quantitative real-time polymerase chain reaction

Before interventional therapy began, 3–5 ml peripheral blood was collected from each patient using EDTA-K_2_ anticoagulation tube (Nantong Wei Ning experimental Audio Supplies Co., Ltd, Jiangsu, China). Blood was centrifuged at 2000 rev/min for 5 min and supernatant was transferred to a clean EP tube followed by 13000 rev/min centrifugation for 10 min. The supernatant was then collected in another clean EP tube and stored at –80°C for later use. Primer sequences are displayed in Table 1. RNA was extracted form prepared blood using the mirVana miRNA Isolation Kit (Thermo Fisher Scientific Inc., Waltham, Massachusetts, U.S.A.), and 5 μl of miRNA was used for reverse transcription in 20 μl of reaction system, including 5 × 4 μl of reverse transcription buffer, 0.75 μl of 10 mmol/l dNTP, 1.20 μl of 1 μmol/l *miR-21* reverse transcription primers L, 0.2 μl of 200 U/μl M-MLV Reverse Transcriptase (MBI Fermentas, Republic of Lithuania) and 8.85 μl of diethylpyrocarbonate (DEPC)-treated water (Guangzhou DaHui Biotech Co., Ltd, Guangdong, China). Reaction conditions included water bath at 16°C for 30 min, water bath at 42°C for 30 min, water bath at 85°C for 5 min and heating at 70°C for 10 min. Prism 7000 fluorescence quantitative PCR instrument (Thermo Fisher Scientific Inc., Waltham, Massachusetts, U.S.A.) was applied to detect the expression of *miR-21*, with 20 μl of reaction system including 5 μmol/l upstream primer and downstream primer of *miR-2* (each for 0.16 μl) respectively, 2 × 10 μl of real time PCR buffer containing SYBR Green I fluorochrome (F. Hoffmann-La Roche, Ltd., Basel, Switzerland), 2 μl of cDNA template (Thermo Fisher Scientific Inc., Waltham, Massachusetts, U.S.A.), 0.2 μl of 5 U/μl Taq DNA polymerase (MBI Fermentas, Republic of Lithuania) and 7.48 μl of DEPC-treated water. Reaction conditions then included water bath at 95°C for 3 min, water bath at 95°C for 12 s, water bath at 62°C for 50 s, with 40 cycles in total. U6 was used as internal reference. The specimen with the lowest *C*_t_ of *miR-21* gene was used as a control. Δ*C*_t_ = *C*_t__miR-21_ – *C*_t__U6snRNA_, ΔΔCt = Δ*C*_t__experiment specimen_ – Δ*C*_t__control specimen_. The relative expression of *miR-21* gene was calculated with 2^–ΔΔ*C*^_t_.

**Table 1:** Primer sequences for quantitative real-time PCR

Primer	Sequence
*miR-21* reverse transcription	5′-GTCGTATCCAGTGCGTGTCGTGGAGTCGGCAATTGCACTGGATACGACTCAACT-3′
*miR-21* amplification	
Upstream	5′-GCGCTAGCTTATCAGA-3′
Downstream	5′-GTGCGTGTCGTGGAGTC-3′
U6 reverse transcription	5′-GTCGTATCCAGTGCAGGGTCCGAGGTATTCGCACTGGATACGACAAAAATATG-3′
U6 amplification	
Upstream	5′-CTCGCTTCGGCAGCACA-3′
Downstream	5′-AACGCTTCACGAATTTGCGT-3′

### Interventional therapy

Two days before interventional therapy commenced, patients were orally administrated 100 mg of aspirin enteric-coated tablets (Harbin Pharmaceutical Group Co., Ltd., China) and 75 mg of clopidogrel (Guangzhou Baiyunshan Pharmaceutical Holdings Co., Ltd., China) once per day. Proper interventional therapy methods were chosen according to the pre-operative CTA and magnetic resonance angiography (MRA) images. Ipsilateral anterograde puncture of femoral artery was adopted initially for stenosis or occlusion of shares in the middle, as well as under shallow of superficial femoral artery and popliteal artery. If the lesions were found in common femoral artery or the upper section of superficial femoral artery or the iliac artery was in the need of intracavitary therapy, then converse puncture of lateral femoral artery was conducted for intracavitary therapy. When the puncture conditions of bilateral femoral artery were insufficient, the puncture was conducted through brachial artery. After local anaesthesia and the puncture was performed, a sheath pipe was inserted. Contrast examination of the target artery was conducted through catheter sheath or catheter subsection, then the therapeutic regimen was further assessed. PTA was applied for patients whose vascular lesion length was 3 cm or less. ES was performed in patients with a long disease course or whose residual stenosis after PTA was more than 30%. Thrombus rotary varicotomy was applied in patients with complete occlusion or whose arterial lesion length was longer than 10 cm. After interventional therapy, patients were subcutaneously injected with 4000 U of low molecular heparin (Guangzhou Baiyunshan Pharmaceutical Holdings Co., Ltd., China), treated with anticoagulant therapy (1/12 h) for 3 days, administered with clopidogrel (75 mg/day) for half year and aspirin (Guangzhou Baiyunshan Pharmaceutical Holdings Co., Ltd., China) (100 mg/day) indefinitely. During the whole process, different types of threads were chosen according to different therapy approaches and forming methods. Ev3 NanoCross and Bantam saccules were adopted for PTA microballoon. As for holders, ev3 ProtegeEverFlex and BARD LifeStent XL were applied. Successful interventional therapy was considered to have reduced lesion blood vessel lumen to <30% with no obvious artery dissection or serious complication, otherwise treatment was considered a failure.

### CAT analysis, ABI measurement and inclusion criteria of vascular restenosis

Follow-up was conducted with subsequent hospital visits and by phone, observing patients’ claudication symptoms and inspecting arteriopalmus. At the end of the sixth month post-surgery, CAT analysis was performed to detect ABI. Based on the result of treatment, all patients were re-divided into restenosis and non-restenosis groups. Effective standard after therapy was as follows: ABI increased 3 days after interventional therapy; symptoms such as intermittent claudication of lower limbs and rest pain were alleviated continuously and anabrosis was completely healed. Otherwise, treatment was regarded as ineffective. Restenosis standard after therapy: conforming to the effective standard, but ABI of the last follow-up was lower than that of prior treatment and CAT showed that the degree of lower limb artery stenosis was greater than that of prior treatment in some patients.

### Statistical analysis

Data were analysed using statistical package for the social sciences (SPSS) version 20.0 (SPSS Inc.; Chicago, IL, U.S.A.). Measurement data were displayed as mean ± S.D. ($\bar{\text{x}}$ ± s). The differences between the two groups were analysed with *t* test while the differences of more than two groups were analysed with variance analysis. Categorical data were expressed as ratio or percentage and chi-square test was conducted. Receiver operating characteristic (ROC) curve was used to estimate the predictive value of *miR-21*. Logistic regression was applied to analyse the risk factors. *P*<0.05 was regarded as statistically significant.

## Results

### Baseline characteristic comparisons of LEAOD patients between restenosis and non-restenosis groups

As shown in Table 2, the mean age of patients in the restenosis group was higher than that in the non-restenosis group, and the ratios of patients with diabetes and hypertension were also higher in the restenosis group (all *P*<0.05). More patients of stage III and IV were found in the restenosis group than in the non-restenosis group (*P*<0.05), whereas no significant differences in age, BMI, course of disease, smoking, coronary heart disease, myocardial infarction or hyperlipidaemia proportion were found in the two groups (all *P*>0.05).

**Table 2:** Baseline characteristics of LEAOD patients between the restenosis and non-restenosis groups

Characteristic	Restenosis group (*n*=79)	Non-restenosis group (*n*=327)	*P*
Male	54 (68.35%)	251 (76.76%)	0.147
Female	25 (31.65%)	76 (23.24%)	
BMI (kg/m^2)^	23.11 ± 2.86	22.62 ± 2.31	0.108
Age (year)	62.02 ± 19.97	58.13 ± 9.98	0.014
Course of disease (year)	3.16 ± 0.63	3.05 ± 0.43	0.069
Fontaine staging			
II	19 (24.05%)	155 (47.40%)	
III	45 (56.96%)	162 (49.54%)	<0.001
IV	15 (18.99%)	10 (3.06%)	
Smoking	54 (68.35%)	215 (65.75%)	0.693
Diabetes	50 (63.29%)	76 (23.24%)	<0.001
Hypertension	50 (63.29%)	118 (36.09%)	<0.001
Coronary heart disease	15 (18.99%)	62 (18.96%)	1.000
Myocardial infarction	15 (18.99%)	72 (22.02%)	0.648
Hyperlipidaemia	14 (17.72%)	64 (19.57%)	0.874

### Expression of* miR-21* between restenosis and non-restenosis groups

**Figure 1:**
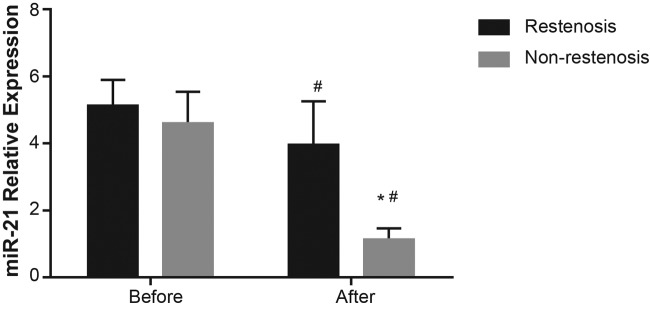
Comparison of *miR-21* expression in restenosis and non-restenosis groups before and after interventional therapy ^#^ refers to *P*<0.001 when compared with levels before interventional therapy; * refers to *P*<0.001 when compared with postoperative restenosis group.

The relative expression of *miR-21* in both the restenosis group and the non-restenosis group after therapy was lower than that before therapy (*P*<0.05). In comparison with the postoperative restenosis group, *miR-21* levels in the postoperative non-restenosis group were significantly decreased (*P*<0.05) (Figure 1).

### The predictive value of *miR-21* on vascular restenosis after interventional therapy

**Figure 2:**
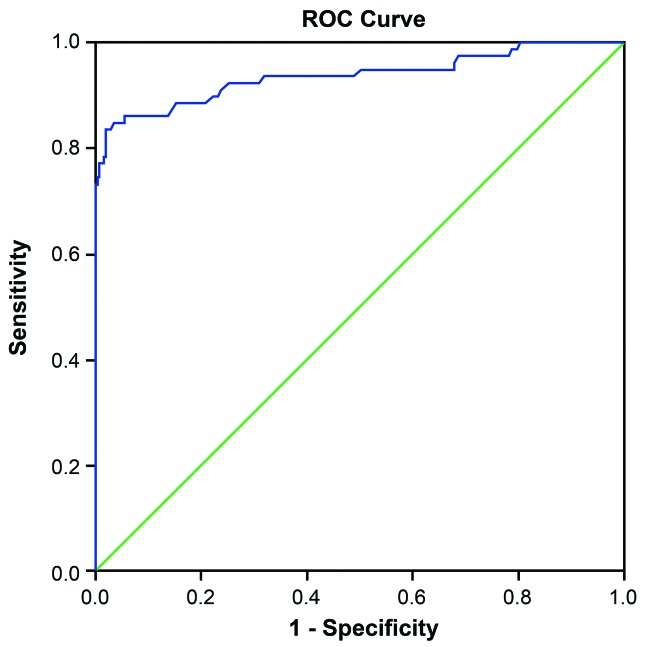
ROC curve of predictive value of *miR-21* for vascular restenosis after interventional therapy Note: ROC, receiver operating characteristic.

The effect of *miR-21* on diagnosing restenosis is presented in Figure 2. The under area of ROC curve was 0.938 (95% confidence interval (CI): 0.898–0.977). The Youden index was 0.817 and the sensitivity and specificity were 83.5% and 98.2% respectively.

### Association between *miR-21* expression and clinicopathological features in restenosis and non-restenosis groups

As shown in Table 3, in the restenosis group* miR-21* expression was correlated to age, diabetes and hypertension. Further, patients with diabetes, hypertension or of older age were found to show higher *miR-21* expression than patients with diabetes, hypertension or of a younger age (all *P*<0.05). However, *miR-21* expression in the restenosis group was not associated with Fontaine staging, smoking, coronary heart disease or hyperlipidaemia (all *P*>0.05). In the non-restenosis group, there was no significant association among the *miR-21* expression and age, diabetes, Fontaine staging, smoking, hypertension, coronary heart disease or hyperlipidaemia (all *P*>0.05).

**Table 3:** Association between *miR-21* expression and clinicopathological features of LEAOD patients in the restenosis and non-restenosis groups

Feature	Restenosis group (*n*=79)				Non-restenosis group (*n*=327)			
	Case	*miR-21*	*t/χ*^2^	*P*	Case	*miR-21*	*t/χ*^2^	*P*
Age (year)								
<50	30	2.73 ± 0.84			132	1.15 ± 0.30		
50–70	27	4.21 ± 0.63		<0.001	164	1.16 ± 0.32		0.104
>70	22	5.34 ± 0.84			31	1.03 ± 0.34		
Fontaine staging								
II	19	3.91 ± 1.24			155	1.17 ± 0.32		
III	45	3.98 ± 1.39		0.982	162	1.12 ± 0.32		0.372
IV	15	3.96 ± 1.25			10	1.14 ± 0.11		
Smoking								
Yes	54	4.05 ± 1.34			215	1.13 ± 0.31		
				0.382				0.178
No	25	3.77 ± 1.26			112	1.18 ± 0.33		
Diabetes								
Yes	50	4.20 ± 1.40			76	1.18 ± 0.27		
				0.036				0.229
No	20	2.51 ± 0.86			257	1.13 ± 0.33		
Hypertension								
Yes	50	4.69 ± 0.93			118	1.18 ± 0.36		
				<0.001				0.101
No	29	2.70 ± 0.84			209	1.12 ± 0.29		
Coronary heart disease								
Yes	15	3.87 ± 1.29			62	1.11 ± 0.42		
				0.773				0.374
No	64	3.98 ± 1.33			265	1.15 ± 0.29		
Hyperlipidaemia								
Yes	14	3.57 ± 1.23		0.219	64	1.18 ± 0.31		0.260
No	65	4.05 ± 1.33			263	1.13 ± 0.32		

### Logistic regression analysis for risk factors of vascular restenosis after interventional therapy

There was no correlation between age, hypertension or Fontaine staging with the occurrence of postoperative restenosis (all *P*>0.05). However, diabetes and *miR-21* expression were indicated to be the risk factors of postoperative restenosis (all *P*<0.05) (Table 4).

**Table 4:** Logistic regression analysis for risk factors of vascular restenosis after interventional therapy

Factor	β	S.E.M.	Wald	*P*	OR (95% CI)
Age	0.018	0.018	0.992	0.319	1.018 (0.982–1.056)
Hypertension	–3.753	2.619	2.053	0.152	0.023 (0.000–3.977)
Diabetes	1.924	0.911	4.467	0.035	6.851 (1.150–40.815)
Fontaine staging	1.017	0.976	1.087	0.297	2.766 (0.409–18.723)
*miR-21* expression	6.297	1.297	23.578	<0.001	542.901 (42.744–6895.482)

OR, odds ratio.

## Discussion

As a common manifestation of systemic atherosclerosis in the limbs, the incidence of LEAOD has undergone a significant increase in recent years, and an interventional treatment called PTA has arisen as a promising treatment for LEAOD [3,5]. However, there are still some complications after interventional treatment, particularly vascular restenosis. An overreaction of biological responses to injury, vascular restenosis is triggered by intimal hyperplasia and constrictive wall recoiling. Although recoil may be minimized by stent placement, there is still an inherent chance of approximately 25% whereby in-stent restenosis is caused by intimal hyperplasia [17]. The present study proposes that there is a correlation between circulating *miR-21* and vascular restenosis after LEAOD interventional treatment. Our study supports the view that diabetes and the expression of *miR-21* are the major risk factors influencing vascular restenosis after interventional treatment. Considering the expression of *miR-21* as an important indicator in predicting vascular restenosis after LEAOD interventional treatment can provide the basis for the development of LEAOD targeted treatment.

In the present study, we found that the relative expression of* miR-21* in the restenosis group was significantly higher than that of the non-restenosis group. Ji et al. [16] showed that *miR-21* is one of the up-regulated miRNAs in the vascular wall after balloon injury and promotes vascular smooth muscle cell proliferation through activation of Akt and Bcl-2 accompanied with inhibition of phosphatase and tensin homologue. Modulating aberrantly elevated *miR-21* levels has a significant inhibiting effect on neo-intimal lesion formation, and the implementation of miRNAs such as with an anti-*miR-21*-coated stent, effectively prevents in-stent restenosis [16,18,19]. There are many risk factors for the development of restenosis after interventional treatment in LEAOD patients, such as non-specific gene transferred to other cell types, lack of regulation of gene expression, induction of an inflammatory response and the resulting uncontrolled level of growth factor [20]. It has been shown that vascular endothelial injury and inflammatory cytokines including IL-6 and IL-10 matrix metalloproteinases are related to LEAOD postoperative restenosis. Further, the expression of inflammatory cytokines in LEAOD restenosis is higher than that in normal vascular tissues. Iliopoulos et al. [21] have shown that the high expression of inflammatory factors such as IL-6 leads to increased expression of miR-21. Moreover, PTEN, as one of the target genes of *miR-21*, has been proved to influence a great number of cellular processes [22,23] and can inhibit the proliferation of vascular smooth muscle cells and reduce the incidence of restenosis [21]. Furthermore, with an inverse relation between elevated plasma lipids and endothelial-healing progression, lipid metabolism is considered as a risk predictor of restenosis [24]. Further to well-established functions of miRNAs in the regulation of cell activities and tumorigenesis, miRNAs have also been shown to participate in the regulation of lipid metabolism, which affects the development and progression of several lipid metabolism-related diseases such as atherosclerosis [25].

In addition, we also found that the proportions of patients with diabetes and hypertension, and those of greater age were significantly higher in the restenosis group than those in the non-restenosis group. Olin and Sealove [26] have reported that the prevalence of peripheral atherosclerosis disease is higher in patients with hypertension than in non-hypertensive patients; diabetic patients have a high morbidity and a rapid and severe disease progression; and aging is closely related with high LEAOD morbidity and postoperative complications [26], all of which support our results indirectly. In addition, Faglia et al. [27] and Saqib et al. [28] all indicate that interventional treatment for diabetic LEAOD has a good short-term effect but shows a high risk of restenosis after and that the metabolic disorder of chronic diabetes may be a cause of restenosis, in accordance with the results of the present study. Moreover, He et al. [29] suggest that circulating miRNAs such as *miR-21*, *miR-143* and *miR-145* are significantly correlated with the occurrence of in-stent restenosis and that miRNAs can serve as potential non-invasive biomarkers for in-stent restenosis after percutaneous coronary intervention surgery [29]. Binary logistic regression analysis further confirmed that the expression levels of *miR-21* and diabetes were risk factors for restenosis after interventional treatment.

As a conclusion, *miR-21* expression is a risk factor influencing the occurrence of vascular restenosis after interventional treatment and therefore *miR-21* can function as an indicator and a promising future diagnostic and therapeutic target for personalized medicine. However, the specific monocular mechanisms of *miR-21s* involvement in vascular restenosis after interventional treatment needs to be further elucidated and a longer follow-up study needs be performed to better assess the predictive value of *miR-21* as a biomarker, and would help us to fully understand the clinical treatment and prognosis judgment when treating LEAOD patients.

## Abbreviations

ABI: ankle-brachial index
AHA: American Heart Association
BMI: body mass index
CI: confidence interval
CTA: computed tomography angiography
DEPC: diethylpyrocarbonate
EP: eppendorf
LEAOD: lower extremity arterial occlusive disease
PTA: percutaneous transluminal angioplasty
ROC: receiver operating characteristic
